# Window into the Brain: In Vivo Multiphoton Imaging

**DOI:** 10.1021/acsphotonics.4c00958

**Published:** 2024-12-24

**Authors:** Shahrzad Latifi, A. Courtney DeVries

**Affiliations:** † Department of Neuroscience, Rockefeller Neuroscience Institute, West Virginia University, Morgantown, West Virginia 26506, United States; ‡ Department of Medicine, West Virginia University, Morgantown, West Virginia 26506, United States

**Keywords:** multiphoton microscopy, brain, mesoscale brain networks, in vivo calcium imaging, volumetric imaging, miniature microscopy, three-photon imaging

## Abstract

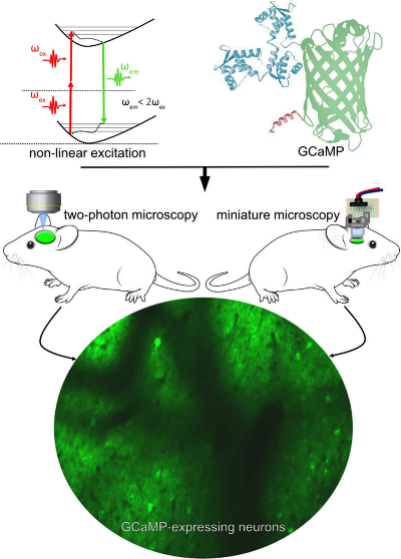

Decoding the principles underlying neuronal information processing necessitates the emergence of techniques and methodologies to monitor multiscale brain networks in behaving animals over long periods of time. Novel advances in biophotonics, specifically progress in multiphoton microscopy, combined with the development of optical indicators for neuronal activity have provided the possibility to concurrently track brain functions at scales ranging from individual neurons to thousands of neurons across connected brain regions. This Review presents state-of-the-art multiphoton imaging modalities and optical indicators for in vivo brain imaging, highlighting recent advancements and current challenges in the field.

## Introduction

One of the fundamental goals in modern neuroscience is to elucidate whether behavior is derived from brain activity. Addressing this challenge requires deciphering signaling and information flow among neural elements within multiscale brain networks that are highly complex and spatiotemporally diverse. Imaging tools have evolved over the past several decades to provide a readout of the activity of neural elements on different scales. Imaging modalities such as magnetic resonance imaging (MRI),^1^ positron emission tomography (PET),^2^ and intrinsic signal optical imaging (IOSI)^3^ have been widely used to track activity of brain networks at macroscale, mainly based on the activity-dependent changes in blood flow. These noninvasive methodologies offer cross-species applications from preclinical studies in experimental models to translational research in the human brain and have provided essential insights into normal and diseased brain function over the past decades. However, the information they provide is derived from indirect measurements of neuronal activity and is limited by the relatively low temporal and spatial resolution. Thus, they do not allow targeting of lower scale neural circuits that are thought to subserve cognition and complex behaviors.^4^

**Figure 1:**
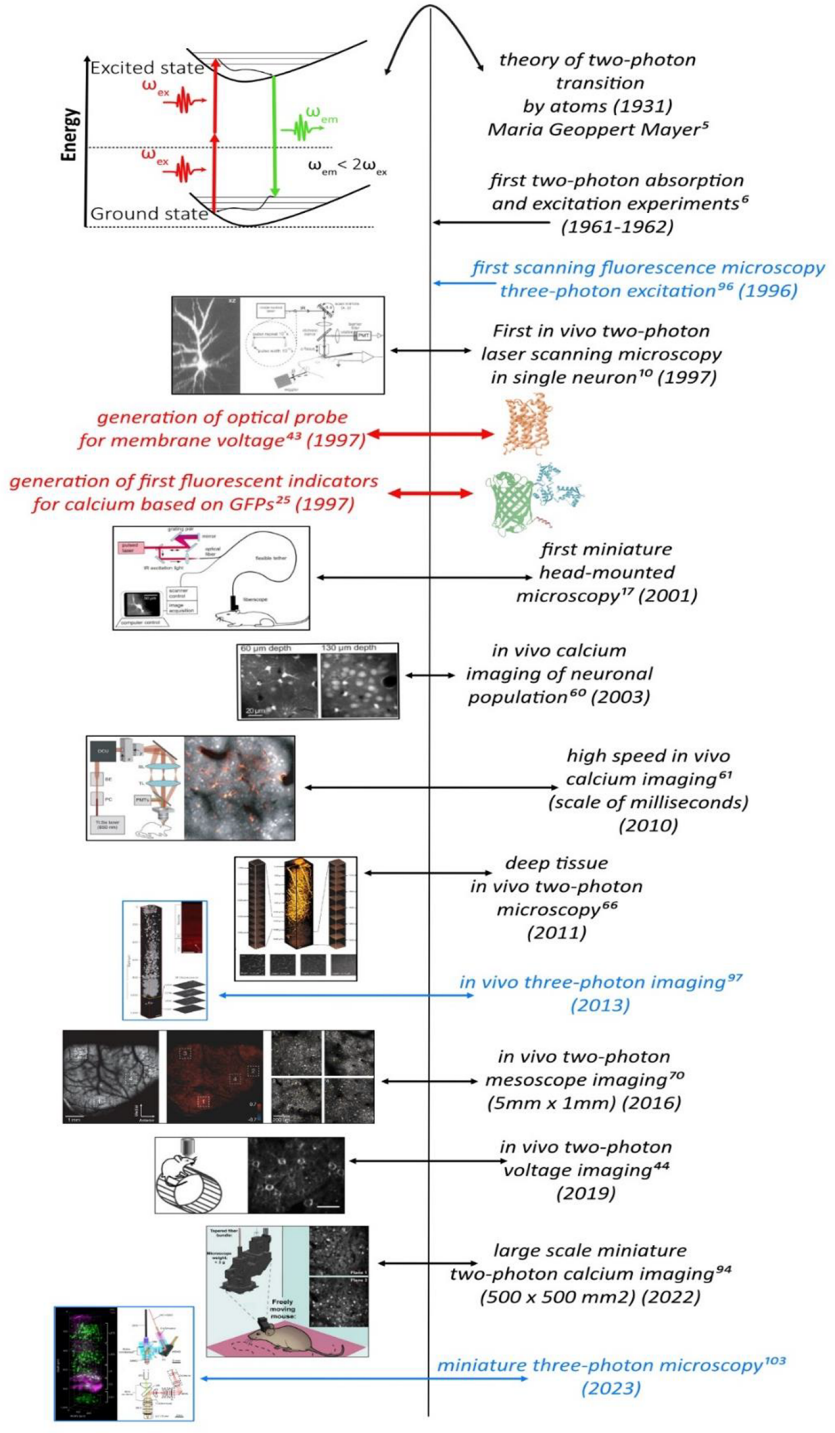
Historical timeline and milestones of in vivo 2P and 3P microscopy from the theory of the 2P transition to the development of miniature 3P microscopy.

Physicist Maria Goeppert Mayer’s introduction of “the theory of two-photon absorption by atoms” in her 1931 doctoral thesis paved the way for multiphoton microscopy.^5,6^ This technology offers the possibility of depth penetration and optical sectioning in scattering living tissue, including the brain, revolutionizing the study of the neural underpinnings of behavior (Figure 1).^7^ More recently, novel optical imaging techniques, in combination with the development of molecular probes that couple neural signals to fluorescence, have promoted the decoding of brain networks functions at lower scales (cellular and circuits).^8,9^ These techniques enable high-resolution imaging of thousands of genetically identified individual brain cell types, from neurons to glia, in the brains of behaving animals and provide an immense amount of information on brain function across days and months.^7^

Since the development of the first in vivo two-photon (2P) scanning fluorescence microscope,^10^ innovations in multiphoton microscopy have produced rapid and substantial technological advances. These include development of high-speed laser scanning (such as the use of acousto-optic deflectors that enable a faster laser-steering) and multibeam technologies, space-division multiplexing techniques, three-photon (3P) excitation, and expansion of field of view (FOV) that resulted in the emergence of imaging at the mesoscale.^11−16^ More recently, innovation of miniature microendoscopy has bolstered the ability of decoding behavior at circuits level with increased flexibility in behavioral assessments.^17,18^ Alongside advances in multiphoton imaging modalities, the generation of optical indicators such as genetically encodable fluorescent indicators of calcium (Ca^2+^), voltage, and neurotransmitters, and more recently, the creation of transgenic lines expressing optical indicators, have rendered the possibility of tracking micro- to mesoscale brain networks in specific cell types or even in specific subcellular domains.^8,19^

This Review summarizes the novel multiscale in vivo brain imaging approaches that can be used in head-fixed or head-mounted freely moving animals. We first introduce novel fluorescent indicators of neural activity, their current delivery platforms from viral to transgenic methods, and their challenges in detecting network dynamics. We then focus on advances and barriers among in vivo 2P imaging modalities by providing an overview of mesoscopic, volumetric, and microendoscopic imaging. Lastly, we present applications of in vivo 3P microscopy and its ongoing developments in brain network imaging.

## Genetically Encoded Fluorescent Indicators of Neural Activity: Viral Vectors and Transgenic Mouse Lines

Over the past two decades, brain science has been propelled forward by the development of several distinct genetically encoded fluorescent indicators that are able to translate neural activity into fluorescence signals.^20^ These indicators enable monitoring of intracellular calcium (Ca^2+^) dynamics, transmembrane voltage, and extracellular transmission, such as vesicle release, and thus provide information about brain function at ensemble (a population of cells), cellular, and subcellular levels that other *in vivo* imaging tools, such as MRI cannot.^8,19^ These indicators generally consist of a sensing domain and a fluorophore; their excitation and emission spectra rely on the chemical properties of their individual fluorophore, and they shift their spectral range as a function of atomic conformation based on their sensing domain responses to the environmental changes.^21,22^ For example, calcium indicators undergo conformational shifts in response to changes in (intracellular) Ca^2+^ concentrations upon binding to ionic Ca^2+8^. Likewise, voltage indicators respond to changes in local electric fields, which result in fluorophore configuration that can provide a readout of transmembrane voltage.^19^ Furthermore, compared to previously used synthetic dyes, which required delivery through cell permeabilization methods such as whole-cell patchclamp or acute bulk loading, that could damage cells, genetically encoded probes offer selective yet unbiased labeling of brain cell types with less damage.^23,24^ Therefore, because these indicators are genetically encoded and can be expressed in specific cell types, neural dynamics can be monitored longitudinally in the context of crucial events such as learning or CNS disease progression.

### Genetically Encoded Calcium Indicators (GECIs)

**Figure 2:**
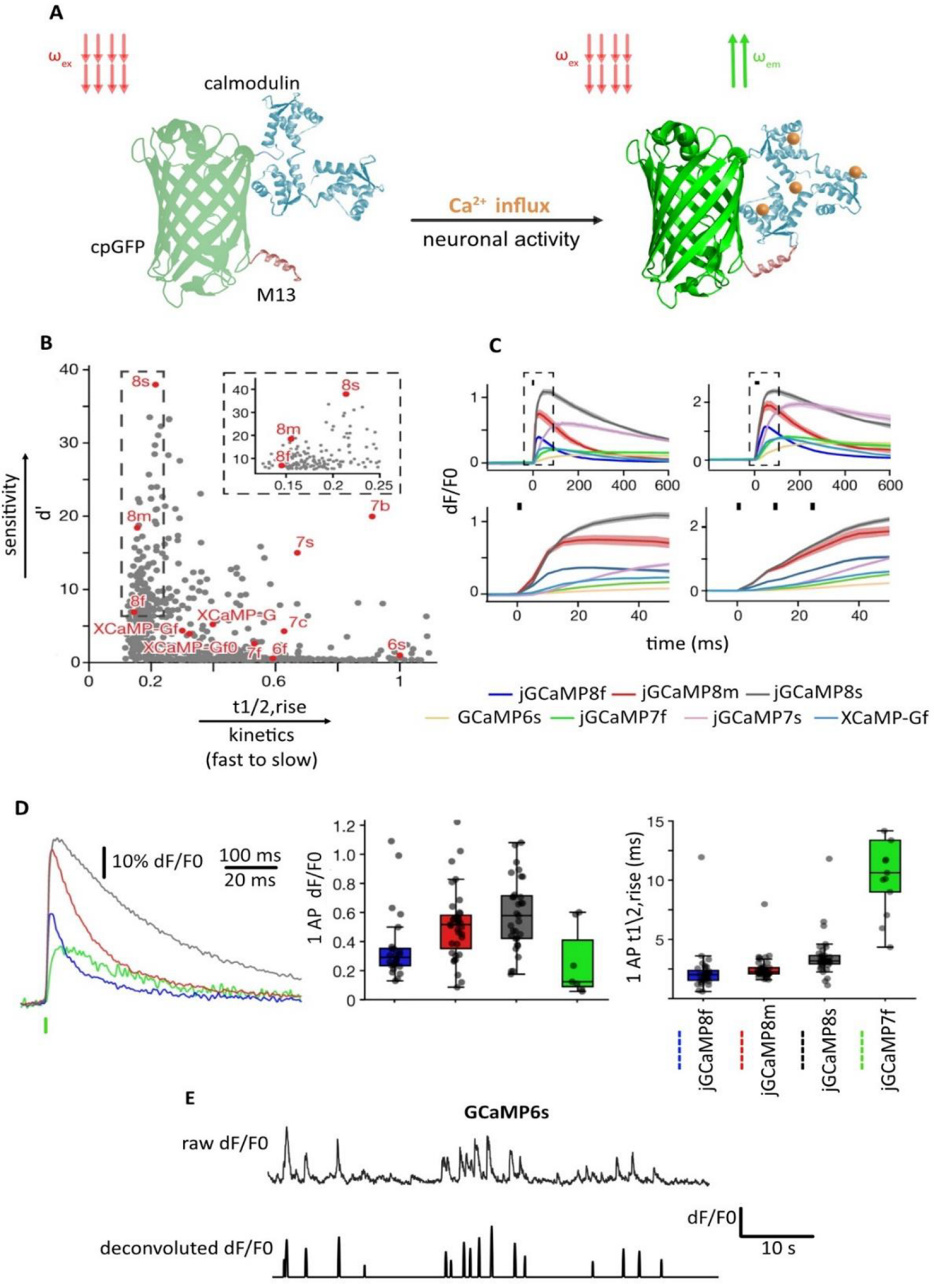
GECI characteristics and functions upon neuronal activity. (A) GECI consists of a circularly permuted green fluorescent protein (cpGFP), calmodulin calcium sensing protein, and a myosin light chain kinase (M13) peptide. Upon neuronal activity (e.g., action potential) and calcium influx in neuron expressing-GECI (e.g., GCaMP6), calmodulin binds to Ca^2+^, resulting in binding to M13 and a conformational change in the fluorophore causing emission of photons from GFP at specific spectra. (B) GCaMP variant kinetics (*t*_1/2_, rise) and sensitivity (*d*′) in a primary neuronal culture. The top right inlaid demonstrates a zoomed-in view of jGaMP8 variants (s, m, and f). The complete multiparameter scatterplots information can be found in ref (30). (C) GCaMP variant calcium transient responses (dF/F0) to 1 and 3 action potentials in neuronal culture. The lower panels show a zoomed view of kinetics from indicated boxes for each GCaMPs. (D) Left: jGCaMP variant average dF/F0 response extracted from single action potentials and aligned to the peak of the action potential (green bar). Middle and right: properties of fluorescence fluctuations in jGCaMP variants in response to single action potentials. (D) is based on the data extracted from simultaneous in vivo electrophysiology and 2P imaging in the mouse V1. (E) An example of traces from primary motor cortical neuron expressing GCaMPs; top panel indicates raw dF/F0 signal, and the lower panel shows an extracted signal after the application of deconvolution algorithm in the top panel. AP: action potential. (B–D) Adopted with permission from ref (30). Copyright 2023 The Authors licensed under a CC-BY Creative Commons Attribution 4.0 License.

The most commonly used optical indicators of neuronal activity in neuroscience are GECIs (Figures 1 and 2). The GECIs are chimeric proteins generally composed of two domains: a calcium-binding domain and a fluorophore. For example, one of the most common used GECI proteins, GCAMP family, consist of a circularly permuted GFP that is fused to calcium-binding protein calmodulin and a M13 myosin light-chain kinase sequence (GCaMP; Figure 2A).^20,25^ Upon binding to Ca^2+^, GCaMP increases its intensity of green fluorescence during activation of the GCaMP-expressing neuron (Figure 2A).^26^ Calcium is the most common intracellular messenger in neurons, with unique roles in neurotransmitter reception and membrane depolarization. In most neurons, resting state intracellular Ca^2+^ levels are very low, but Ca^2+^ influx spikes during multiple neuronal events. For example, voltage-gated calcium channels in dendritic spines allow Ca^2+^ influx upon local dendritic spiking and in excitatory neurotransmission, NMDA-type glutamate receptors mediate a Ca^2+^ increase in synaptic spines up to 1 μM.^27^ Moreover, during action potential propagation in neuron, voltage-gated calcium channels allow Ca^2+^ entry, resulting in membrane depolarization and a somatic Ca^2+^ increase (150 nM within 10 ms) that persists with a half decay time of 50 ms.^26,28^ In addition, calcium-permeable AMPA receptors can also be involved in some forms of synaptic calcium signaling that represent an alternative source for activity-dependent calcium entry, facilitating the initiation of synaptic plasticity.^29^

Since the generation of the first GCaMP, several variants have been developed with distinct molecular properties.^30^ Recent variants have distinct kinetics and strong fluorescence intensity that provide more accurate reporting of intracellular Ca^2+^ and signal-to-noise ratio (SNR).^30,31^ In fact, the sensitivity and kinetics of GECIs are among the important factors that alter their capabilities in responding to neuronal activity and shaping fluorescence waveforms. For example, GECI sensitivity to Ca^2+^ fluctuations (related to a single spike) increases based on its strength in binding to Ca^2+^, however, this can result in longer decay time in fluorescence waves. Thus, early generations of GECIs had generally stronger intensity, but a greater decay in optical waveforms than more modern GECI models (Figure 2B).^30^ For example, GCaMP6s have a rise time of 50–200 ms and a decay time of 150–500 ms for a fluorescence time course related to a single spike, whereas the new GCaMP8 family has substantially shorter rise and decay times of 10 and 50–200 ms, respectively (Figure 2B–D).^30,32^ Using more recent GCaMP variants, such as jGCaMP7 and jGaCMP8, a single action potential can be detected with 3- to 10-fold rises in fluorescence intensity. The most recent variant, X-CaMP-GF, offers the promise of reporting single action potentials at firing rates up to 20 Hz (Figure 2B–D).^30,33,34^ While different subtypes of neurons feature distinct Ca^2+^ physiology, such as intracellular Ca^2+^ buffering that could also affect the GECI temporal resolution of Ca^2+^ reporting,^8^ the X-CaMP family of GECIs are able to reliably resolve isolated spikes, including neurons with relatively high baseline firing rates, such as fast spiking interneurons (Figure 2B–D).^30,33^ In addition, the emergence of newly developed red spectral GECI variants, including RCaMP, X-CaMP-R, and R-GECO, provides the possibility of simultaneous tracking of neuronal activity among various neuronal subtypes expressing distinct indicators with different spectral ranges.^33,35−37^

#### Calcium Dynamics, Neuronal Activity, and Considerations

GECIs can be used as a reliable indicator of Ca^2+^ transients during multiple neuronal events based on their photophysical features that are limited by the inherent quantum mechanical randomness of photon emission and detection (Figure 2).^8,26^ Upon binding of Ca^2+^ ions to calmodulin, the protein undergoes a conformational change that results in deprotonation and a fluorescence change of the associated fluorophore. A GECI also can report a reduction in neuronal spiking rate due to the accompanying reduction of depolarization-induced Ca^2+^ influx.^8,19,26^ While several improvements have been made since the development of the first GECIs, they are still generally insensitive to hyperporalization of the cell membrane below its resting potential or subthreshold voltage changes. In their slow versions such as GCaMP6s or GCaMP7s, the timing of sparse action potentials can be indirectly inferred using deconvolution algorithms to exclude the slow decay of Ca^2+^ transients; however, this should be adjusted based on the indicator’s kinetics (Figure 2E).^30,38−40^

### Genetically Encoded Voltage Indicators (GEVIs)

An alternative approach is to track neuronal activity by monitoring the membrane voltage dynamics via GEVIs, which offer higher temporal resolution than GECIs. Transmembrane voltage shows rapid transmission of signals across long distances (millisecond time scale range and varying distances depending on the type of neuron) with various behaviors across multiple time scales.^41−43^ This is influenced by the depolarization and hyperpolarization of synaptic inputs. Furthermore, synaptic activity can result in activation of voltage-gated channel and spike generation, or even transient depolarizations without a spike.^8,19^ These events were reported by GEVIs. In addition, GEVIs can detect axonal spikes as isolated, in trains, or in bursting activity with the frequency higher than 100 Hz.^44^ The ability to differentiate among these various electrical signals of neural activity is advantageous and could provide an immense amount of information about neuronal signaling. However, detection of these signals is challenging, particularly in nonlinear multiphoton microscopy, and requires scanning faster than the standard 2P laser scanning microscopy techniques.^45,46^ For example, detecting high frequency bursting activities requires an acquisition system with high sampling rates (>2 kHz for a single spike). The current versions of GEVIs are generally consisting of a fluorophore and a voltage-sensing domain (VSD). The VSD can be a 4-transmembrane helix voltage sensor domain derived from a voltage-sensing phosphate (DSP) or a microbial rhodopsin.^44,47,48^ For in vivo imaging, VSD-based GEVIs, such as the ASAP family, are more popular compared to opsin-based GEVIs in reporting voltage dynamics in neuronal population and stimulus-evoked single-cell voltage fluctuations due to the weaker voltage-sensitive fluorescence in opsin-based GEVIs.^44,49,50^ It should be noted that a subgroup of rhodopsin-based GEVIs, the electrochromic FRET (eFRET) GEVIs, where the rhodopsin serves essentially as a VSD, show enhanced brightness.^44^ Moreover, GEVI needs to be located within the plasma membrane; thus, the number of indicators that can fit within a two-dimensional membrane is much smaller than the three-dimensional cytoplasm, which means a high concentration of indicators is hard to achieve. In addition, too many GEVIs located inside the membrane may cause clogging and steric effects on the conductance of the membrane receptors and channels.

#### Voltage Dynamics and In Vivo Multiphoton Microscopy

While GEVIs have been widely used to track neuronal activity in vitro, ex vivo, or in transparent experimental models in vivo (such as larval zebrafish, fruit flies, or nematodes), their applications for in vivo mammalian studies (e.g., mice) are challenging. Further optimization is required to enhance membrane expression, voltage sensitivity, and increase signal-to-noise ratio (SNR).^44,47,49^ For example, one of the challenges is to achieve adequate SNR which is intrinsically small for voltage imaging because the signal is coming from proteins that are located in the 2D cellular membrane. This requires an optimized imaging instrument that can generate and sample the maximum photons per time bin.^48^ Thus, approaches such as point-scanning cannot be considered optimal for in vivo voltage imaging and improvements are required in imaging techniques especially in nonlinear excitation methods such as multiphoton microscopy. For example, random access microscopy based on acousto-optic holography could provide voltage imaging of a limited number of cortical neurons in layer V of awake behaving mice.^49^ A more recent study has demonstrated that parallel excitation methods developed for scanless 2P can be used for voltage imaging of barrel cortex neurons in anesthetized mice.^51^ However, the maximum achievable imaging depth in these approaches was limited and did not exceed 250 μm below the cortical surface. Therefore, future advances will ideally provide GEVIs with different spectra for deeper imaging in various cell types and a great number of neurons.

Since their development, both GECIs and GEVIs have provided the possibility of probing functional neuronal circuits with high spatiotemporal resolution.^49^ Despite the indirect measurement of action potential by GECIs, they are currently more often employed in tracking neuronal activity than GEVIs. Greater SNR, specific expression in desired regions of interest or in particular cell types, wider spectral ranges, better suitability to the current multiphoton imaging frame rates, and development of transgenic lines for GECIs, are among several factors that led to the nearly universal use of GECIs. Considering the importance of voltage imaging for the future of brain science, further improvements and innovations are required for both GECIs and GEVIs.

## Expression of Optical Indicators for an In Vivo Brain Study

**Figure 3:**
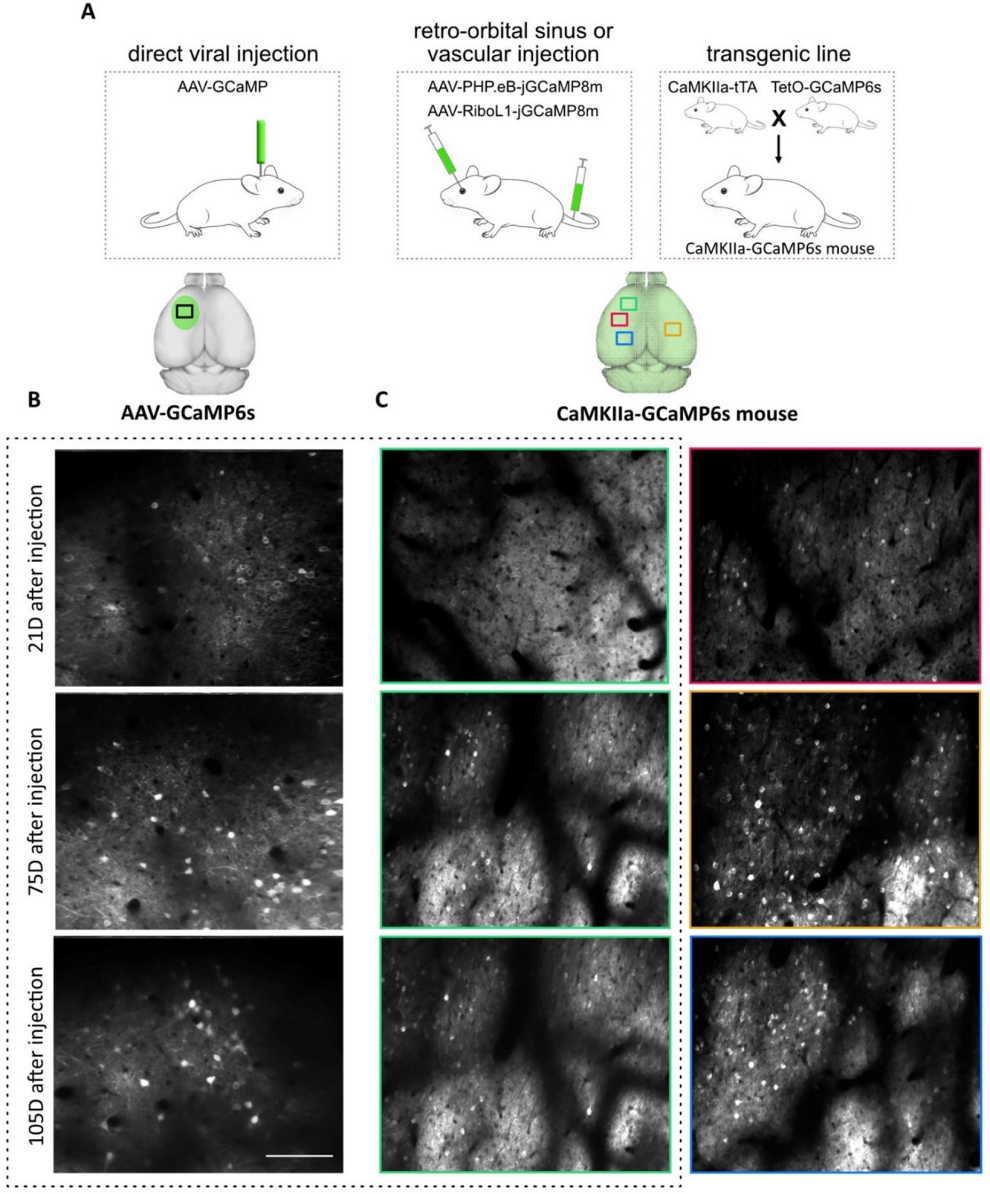
Viral and transgenic methods for labeling neurons with optical indicators. (A) Left: intracerebral viral injection using stereotaxic surgery. The region of GCaMP-expressing neurons is limited to the site of injection. Middle: recently developed adeno-associated virus, AAV-PHP.eB-jGCaMP8 and AAV-RiboL1-jGCaMP8, that cross the blood–brain barrier and enable genetic targeting of neurons across the brain following intravascular virus injection or retro orbital sinus injection. Figure depicts injection of jGCaMP8m into the veil of eye. Right: transgenic line creation (e.g., tetO-GCaMP6s). Expression of calcium indicator in this line is under the control of a tetracycline-responsive promoter element (TRE;tetO). When hemizygotes are bred with another transgenic mouse expressing tetracycline-controlled transactivator protein (e.g., CaMKIIa-tTA), GCaMP6s expression can be regulated. Double positive offspring constantly express GCaMP6s across the brain. (B, C) Example of GCaMP6s-expressing forelimb premotor cortical neurons under viral expression (B) or in transgenic mouse (C, left). Different time points demonstrate an increase in the number of GCaMP6s-overexpressing neurons in viral methods. Time points are calculated based on the time of cranial window implantation alone (C-left) or combined with the GCaMP6s viral injection (B). (C, right) Expression of GCaMP6s in different regions of the brain corresponding to colored boxes (C, top). The red box indicates 2P imaging from primary motor cortical neurons, orange box from somatosensory forelimb, and blue box is from somatosensory hindlimb motor cortex. Scale bar, 150 μm. (B) and (C) images courtesy of S. Latifi.

Delivery of genetically encoded fluorescent indicators into the brain is a crucial step for in vivo imaging application. Two methods are widely used for adequate transgenes expression in the living brain that include a (1) viral approach and (2) transgenic approach (Figure 3). Recombinant adeno-associated viruses (AAVs- 1 to 9) are among the most common vectors involved in the gene delivery of GECI to specific brain regions of interest and can target different subtypes of neurons.^37,52,53^.^52,54^ Compared to other viral vectors, such as lentivirus, the production of AAVs at high titer is less challenging, and they have higher infection rates with lower associated immune responses in the injected animals. For in vivo brain inter-regional or interhemispheric imaging, viral labeling targets neurons through their axonal projections mainly at axon terminals and retrograde transport to neuronal somas.^55,56^ For anterograde labeling, various AAV serotypes, such as AAV1, can be used to trace neurons in an anterograde trans-synaptic approach.^37^ Cre-recombinase AAV vectors can also be used to transduce the expression of optical indicators in specific cell types.^31,37,54^ The second approach in tracing neuron-expressing optical vectors is via the generation of transgenic animals.^52,53,57^ Transgenic approaches offer the benefit of persistent and uniform expression of the optical indicator, allowing longitudinal tracking of neuronal activity. Currently, several transgenic mice, such as Thy1-GCaMP6s, are available that are mainly developed for GECIs expression (Figure 3).^52,53^ Like AAVs delivery, transgenic-based approaches on Cre- or Flp-recombinase expression of optical indicators are also widely used for in vivo imaging of specific neuronal subtypes. For inducing the expression in several classes of distinct cell types, a GECI-expressing line can be crossed with more than one driver line^31,52,56^ (Figure 3).

### Pros and Cons in Current Gene Delivery Platforms

The primary limitations associated with using the viral vector approach are consistency and persistence. AAVs approach offers neuronal expression of a transgene of interest. The transduction rates, however, depend on several factors, including the serotypes of the virus, regions of the brain, and delivery routes. One of the most common delivery methods to achieve and maintain a suitable viral titer in the region of interest is stereotaxic intraparenchymal injection. Since the majority of in vivo multiphoton microscopy necessitates a craniotomy, having a long or multiple separate procedures for viral injection and cranial window implantation can be complicated for the animal and can induce inflammation and neurodegeneration along the injection track.^56,57^ Moreover, AVVs generally diffuse a relatively short distance and thus can be expressed only in limited areas surrounding the injection site. Thus, recording from several fields of view requires multiple injection sites to reach the adequate GECI-expressing neurons/regions. Whereas most AAVs do not cross the blood-brain barrier (BBB), there are other serotypes that reliably cross the BBB and allow for a less invasive GECI delivery. For example, GECIs can be administered through an intravenous (IV) injection into transverse sinus or tail using AAV9 stereotype that can cross the BBB in neonatal mice.^54,56,58^ However, IV injection requires a high concentration of viral vectors and can cause rapid immune responses. Furthermore, it can cause overexpression of GECI due to the young age of animal at the time of injection, and thus may affect the quality of neuronal activity tracking in overexpressed cells (Figure 3).^56^ Recent developments in AAVs GECI delivery are more promising; for example, the new PHP.eB stereotype crosses the BBB in adult mice to express jGCaMP8m through noninvasive retro-orbital sinus injection (Figure 3).^56,58^ Finally, in longitudinal imaging, the overexpression of fluorescent indicators induced by viral vector is one of the major challenges that can limit the time window of recording to several weeks. The overexpression of GECIs protein in neurons can result in longer slow decay, cellular damage, nonphysiological Ca^2+^ transient signal and thus may reduce the quality of recording.

Another less invasive approach is the generation of a transgenic line. In contrast to the viral vector approach, transgenic mice have consistent expression of the fluorescence indicator without a potentially damaging effect on neuronal function. In fact, the uniform and stable expression of GECIs across transgenic animals of the same line provides the possibility of longitudinal and complex imaging studies, as well as functional tracking/analysis of specific neuronal ensembles dynamics over a period of months, and could enhance data reproducibility. The primary limitations of the transgenic mouse approach include the need to maintain colonies (which is expensive and time-consuming) and fewer opportunities to customize compared to AAVs. For example, the most well-characterized GECI transgenics are mainly reporters of the slow version of GCaMP in excitatory circuits (e.g., Teto-GCaMP6s or Thy1-GCaMP6s) and cannot be targeted for subcellular neuronal compartments such as specific axonal projection patterns.^53,57^ Other examples of GECI transgenic lines that have been widely used are TIGRE1.0 and TIGRE2.0, which were generated by Allen Brain. These lines are Cre- and tetracycline transactivator (tTA)-dependent transgenic platforms with a high GECI expression level. While TIGRE2.0 expression level of GECI can be comparable to that of strong promoter-driven AAV vectors, some GECI-expressing, TIGRE-based mouse lines were reported to exhibit significant abnormalities in their brain activity.^52,59^ Another challenge in using GECI transgenic mice is that like other transgenics, their compatibility for use as a disease model or for specific behavioral tasks must be characterized a priori. For example, in experimental stroke studies, their poststroke functional deficits should be first studied and compared to more commonly used mouse strains and wild-type lines. Furthermore, the generation of these lines sometimes require double or triple transgenic crossings that can cause health issues in offspring, such as increased susceptibility to generalized seizures.^59^

With all of the pros and cons, both techniques have been established for in vivo brain imaging studies, providing the possibility of tracking brain functional networks at several scales. Further ongoing developments can optimize these approaches with more specificity in expression, less invasive procedures, and more selectivity in cell types of interest or their spectral range. Moreover, since these approaches are not mutually exclusive, a combinatorial procedure can be used for intersectional targeting or labeling various indicators (e.g., injection of virus in a transgenic mouse for expressing a combination of different GECI indicators).

## In Vivo Two-Photon Microscopy: Advances and Challenges

**Figure 4:**
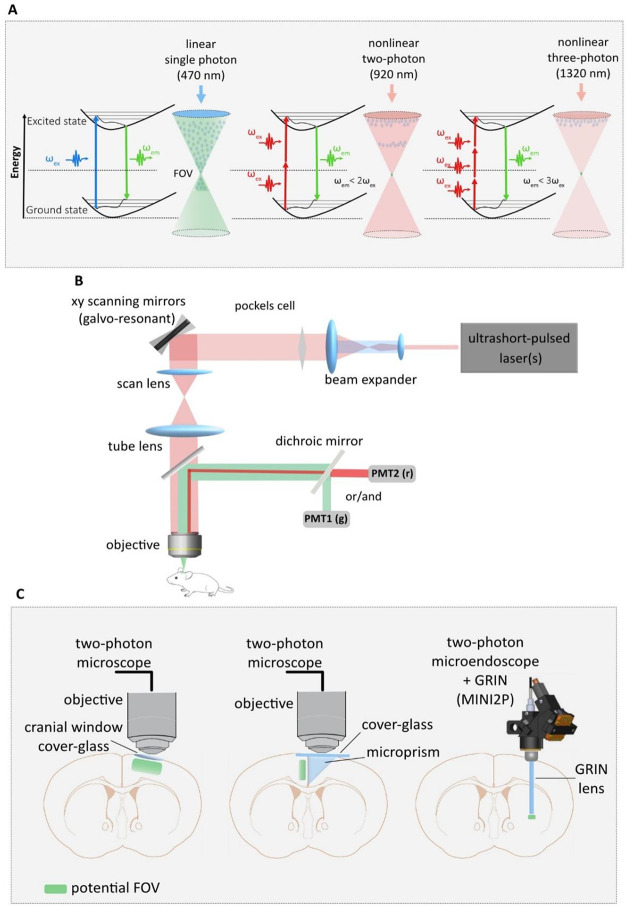
Two-photon imaging of the brain. (A) Nonlinear vs linear excitation. Near infrared is used for 2P or 3P excitation, and visible (blueish) light is used for single-photon excitation. Beam from nonlinear excitation is localized to the vicinity of focal plane, whereas in linear process an entire con of fluorescence light is generated. (B) A conventional in vivo 2P microscope and its compartments: a single wavelength laser is presented as a light source. (C) Left: standard cranial window for imaging superficial layers (e.g., L2/3) of prefrontal cortex under 2P microscopy. Middle: an example of deep layer imaging using a prism under 2P imaging. Right: an example of miniature microscopy (e.g., MINI2P)^94^ using gradient refractive index (GRIN) lens for deep brain imaging in freely behaving mouse combined with 2P excitation and recording.

2P imaging is a nonlinear optical microscopy technique that relies on nonlinear interactions between two near-simultaneous photons and a fluorophore. To induce these nonlinear interactions, a high spatiotemporal density of excitation photons is required, which is achieved through the use of pulsed laser sources that provide temporal confinement of the excitation photons. These interactions result in the transition of the fluorophore from the ground state to the excited state, leading to the emission of fluorescent light (see Figure 4A).^52−55^ In comparison to single-photon fluorescence microscopy, 2P imaging offers the advantage of deep tissue penetration within the intact living brain, enabling the recording of neuronal activity from thousands of cortical neurons to depths of several hundred micrometers below the dura mater.

This crucial advantage is attributed to two key features of the nonlinear process: (1) Scattering and absorption have a significant impact on the imaging depth in biological tissues by attenuating the excitation laser pulse and emitting fluorescence. As photons travel deeper into the tissue, the probability of scattering and absorption increases, resulting in reduced signal intensity and imaging depth.^60,61^ Scattering and absorption effects vary by wavelength; longer wavelengths are less prone to scattering compared to shorter wavelengths, allowing for deeper tissue penetration. Consequently, increasing the excitation wavelength can mitigate the effects of scattering and absorption. In the nonlinear process, excitation occurs through relatively long illumination wavelengths, achieved by combining multiple photons of energy lower than the one required for the corresponding single-photon transition. This effectively reduces light scattering, thereby enhancing the probability of deeper penetration into the brain tissue. (2) The nonlinear excitation process requires simultaneous absorption of two photons from the excitation beam. Thus, the probability of two photon absorption by a fluorophore scales with the square of the incident laser intensity. This is reachable only at the focal plane of the objective lens, confining the signal to the plane of focus. Confinement of the signal to the focal plane is called optical sectioning that significantly reduces out-of-focus signals (reducing photobleaching and phototoxicity; see Figure 4A).^56,57,62^

In 2P excitation, femtosecond lasers (typically 80 to 120 fs) possess the requisite high peak power necessary to maintain low average power to minimize tissue damage. The most commonly used lasers for 2P imaging typically offer tunable wavelengths spanning from 700 to 1020 nm. This enables effective excitation of the majority of genetically encoded fluorescent indicators commonly used in 2P brain in vivo imaging. Newly developed lasers can be tuned to longer wavelengths, exceeding 1,080 nm, and are notably more efficient for the infrared excitation spectrum.^58^ This is specifically beneficial for achieving improved tissue penetration in brain imaging. The latest advancements include single-wavelength fiber lasers operating at 920 or 1060 nm, specifically suitable for optimal imaging of green and red GECIs respectively.^59^ Furthermore, recent studies demonstrated that high-energy, low-repetition rate lasers (from hundreds of kHz to a few MHz) could enhance excitation efficiency and potentially increase penetration depths (while maintaining powers below the damage threshold).^46,51^

In addition to the pulsed laser, a typical 2P imaging setup includes a power modulation system such as a Pockels cell, a beam scanning system, one or more photomultiplier tubes (PMT) for collecting emitted photons from the sample, a mechanical or electrical shutter to control the timing of the laser pulses and fluorescent detection, and an objective (or more than one that can be used simultaneously; see Figure 4B). Currently, several beam scanning systems exist for in vivo 2P imaging. For example, commonly used galvanometer-based mirror scanners (galvos) consist of a pair of lightweighted galvanometric mirrors that are typically mounted on orthogonal axes, and can be rapidly tilted using galvanometers. Another scan system is resonant scanning composed of a mirror that oscillates back and forth along the same line at high frequencies (several kHz). This increases the effective scanning speed and enables fast line scanning of neuronal events such as calcium transients. Some 2P systems combine scanning techniques to achieve optimal imaging results. For example, standard galvanometric scans for *y*-axis can be combined with resonant mirrors for the *x*-axis (galvo/resonant mirror combinations), resulting in scanning of the tissues at a fixed speed rate (8–12 kHz resonance frequency).^63,64^ Based on the applications, other scanning approaches can also be used in 2P microscopy. For example, acousto-optic deflectors (AODs) employ acousto-optic effect to steer the laser beam across the sample with high resolution.^65^ These create targeted path scanning and maximize the time spent on the structures of interest. For efficient scanning, other techniques such as spatiotemporal multiplexing or piezoelectric devices can be combined with scanning approaches.^66^ For example, combining galvo or resonant mirrors with piezoelectric stages provide precise adjustments to the position of the sample in the *z*-axis in 3D imaging.^67^

Currently, the resonant-galvo mirror combination has become the most commonly used scanning approach in 2P GECIs 2P imaging. Among several advantages, this scanning system provides sufficiently fast sampling rates to capture the functional dynamics of GECI-expressing neurons (30 Hz for 512 × 512 pixels). Moreover, the system’s ability to produce several frames per FOV, which can be averaged together, enhances the SNR of the acquired images, however, with the caveat of reducing temporal resolution. Finally, the galvo-resonant mirror approach could offer flexibility in imaging parameters, such as scanning rates, spatial resolution, and field of view.^9^

### Mesoscopic Imaging

Since the development of the first 2P microscope, several improvements have been made to provide the possibility of tracking brain network dynamics from meso- to macroscale levels with single-cell resolution. For example, in conventional 2P microscopy, it is possible to monitor the activity of neuronal ensembles up to a recording FOV of 0.7 mm^2^.^68^ Technological advances have expanded the FOV to 5 mm^2^ (mesoscopic imaging), allowing a simultaneous recording from several regions of the brain in both hemispheres.^12,68^ In building 2P mesoscopic microscopy, a complex interplay among FOV dimension, number of pixels per image, and frame rate should be considered; these parameters often involve trade-offs that impact the quality and efficiency of image acquisition. It should be noted that these trade-offs are also present in standard scanning imaging techniques. Increasing the number of pixels per image enhances spatial resolution. In parallel, higher pixel counts result in larger data volume and longer image acquisition time. A larger FOV at high spatial resolution (larger number of pixels) can reduce the frame rates because more time is needed for registering an image. In parallel, similar FOV can be recorded with higher frame rates but at lower spatial resolution. Thus, 2P mesoscopic recording with large FOV (>2.5 mm^2^) is still challenging since it affects other features of image acquisition and requires development of faster laser-scanning. An alternative method to record from multiple brain regions (to enlarge the FOV) is spatiotemporal multiplexing that involves splitting the excitation beam into multiple beamlets.^66,69,70^ In this approach, each beamlet has a specific temporal delay relative to the others, causing emitted photons to be separated based on their detection time. This enables the simultaneous capture of multiple image planes by rapidly switching the focus of the excitation laser beam between different depths within the specimen. It is important to note that the delayed pulses are generated within the pulsed source period, meaning that they occur within the time frame of each laser pulse. This temporal relationship allows for effective multiplexing of the fluorescence signals. Additionally, adjusting the repetition rate of the laser source can impact the number of delayed pulses generated within each pulse period. Lowering the repletion rate can result in an increased number of delayed pulses, thereby enhancing the multiplexing capability. However, it should be noted that this technique can be challenging with commonly used lasers (>80 MHz fs pulsed), since the number of acquisitions (pulse trains) is limited due the fluorescence lifetime of the activity indicator and the laser pulse repetition rates.^12^ In recent studies, this limitation is addressed by decreasing the repetition rates of the laser to increase the number of pulse trains.^71^ Another strategy in achieving large FOV is 2P frequency-division multiplexing that generates amplitude modulation of femtosecond laser pulses in MHz, however, this technique requires further optimization before being suitable for most in vivo brain studies.^72^

The trade-off between scanning speed and signal is not limited to mesoscopic imaging; a higher frame rate can increase image pixel size that results in fewer 2P excitation events and thus fewer emitted photons from fluorescent indicator. In general, these parameters can be controlled by the acquisition software of a 2P setup and should be adjusted based on the experimental designs and conditions, such as size of FOV, type of fluorescent indicators and the level of expression, depth, and sometimes duration of recording.^73−75^ In addition, these parameters sometimes need to be changed during the course of an experiment, for example, by an increase in the number of overexpressing GECI-neurons.

### Volumetric 2P Microscopy

Advances in 2P microcopy have also offered noninvasive three-dimensional (3D) recording in deep brain tissue. Initial in vivo brain studies used piezoelectrically induced mechanical vibration of objective and smart 3D line-scanning that allowed calcium imaging from hundreds of neurons in volumes up to 250 μm side length and at 10 Hz.^65,76,77^ Further approaches have been developed providing high-speed 3D monitoring of brain networks at larger volume; for example, Bessel-focus scanning elongates the excitation of the laser beam and records from a large volume by extending the depth of field.^78,79^ Most recently, a 2P synthetic aperture microscopy enables long-term continuous 3D imaging by capturing multiple angular projections of the entire 3D volume with needle like beams instead of previously used point-scanning with high numerical aperture.^80^ With all the developments in 2P 3D imaging over the past decade, several challenges, such as low axial and temporal resolution,^81^ remain to be addressed. Thus, further improvements are required for long-term high-speed, high-resolution 3D imaging in the living brain.

### Deep Brain 2P Imaging

The current 2P imaging is restricted to the superficial brain regions (typically cortical regions) due to the attenuation of excitation and emission lights caused by tissue scattering at deeper brain layers. While using optical indicators with excitation spectra within the infrared spectrum can reduce the scattering effect and thus improve penetration, the recording depth is still limited to ∼800 μm from the surface of the brain. One traditional method is to use a microprism that can be implanted in the fissures between hemispheres to assess neuronal activity in medial brain structures such as the prefrontal cortex. This method laterally targets the region of interest, reflecting photons at 90°, and cannot go deeper than ∼1.2 mm due to optical constraints and tissue properties.^82,83^ Other techniques target deeper brain regions, such as hippocampus or amygdala, by aspirating the cortex and implanting glass canula plugs on top of the region of interest; however, this approach is highly invasive and could alter brain signaling (Figure 4C).^84,85^ The most common approach to image neurons in deep brain regions is microendoscopy.^86,87^ This approach generally contains one or two gradient refractive index (GRIN) lenses that steer photon propagation through gradual variations of optical refractive index.^88,89^ The gradual variation can produce lenses with a flat surface with typical diameters of 350 μm to 2 mm. The lengths of microendoscope varies based on the region of recording (typically from 2 to 10 mm; Figure 4C).^82,89^ While microendoscopes can be used in head-fixed 2P imaging, they are also compatible with other imaging modalities including the newly developed miniature 2P microscopy.^17,90−92^

### Miniature Microscopy

Compared to conventional head-fixed 2P microscopy with limited choices of behavioral tasks, head-mounted miniature microscopy (miniscope) does not provide mechanical constraints for head fixation and can therefore provide recording in freely behaving animal.^92^ This is especially important in experimental models in which network topology is to be evaluated for a specific functional behavioral deficit related to a disorder such as Alzheimer’s disease or stroke, during its onset, progression, or recovery. It should be noted that the current miniature microscopies (if used to target deep brain regions) are considered invasive approaches since the implementation of lenses into deeper brain structures requires aspirations of brain tissues. Thus, a full recovery from potential disruptions of neuronal network activity or structure, as well as changes in animal behavior after the implementation procedure, is recommended.

In the present 2P (and single-photon) miniature microscopy, the FOV is generally fixed over the period of recordings (∼400 μm^2^-FOV and 180 μm deep z-stack).^12,68,92,93^ This provides constant monitoring of network dynamics at the level of a single neuron and escapes challenges in finding the exact coordinate of the recording (FOV). However, the trade-off is that it gives limited access to tracking multiple regions of interest. Another important issue is related to the weight of the head-mounted miniature scope that can interfere with the animal’s movement or distinct behavior during extended recording. Further improvements are required to assess several brain regions in the same animal carrying a lighter miniscope device. In a recent development, MINI2P enables imaging from thousands of neurons with high sampling rate and high signal-to-noise ratio, with capability in deeper z plane recording. By retaining the optical sectioning capacity of 2P excitation, this new development is able to monitor network activity through a 1.3 × 1.3 × 1.6 mm^3^ prism.^94^

## Deep within the Brain: Application of Three-Photon Microscopy

**Figure 5:**
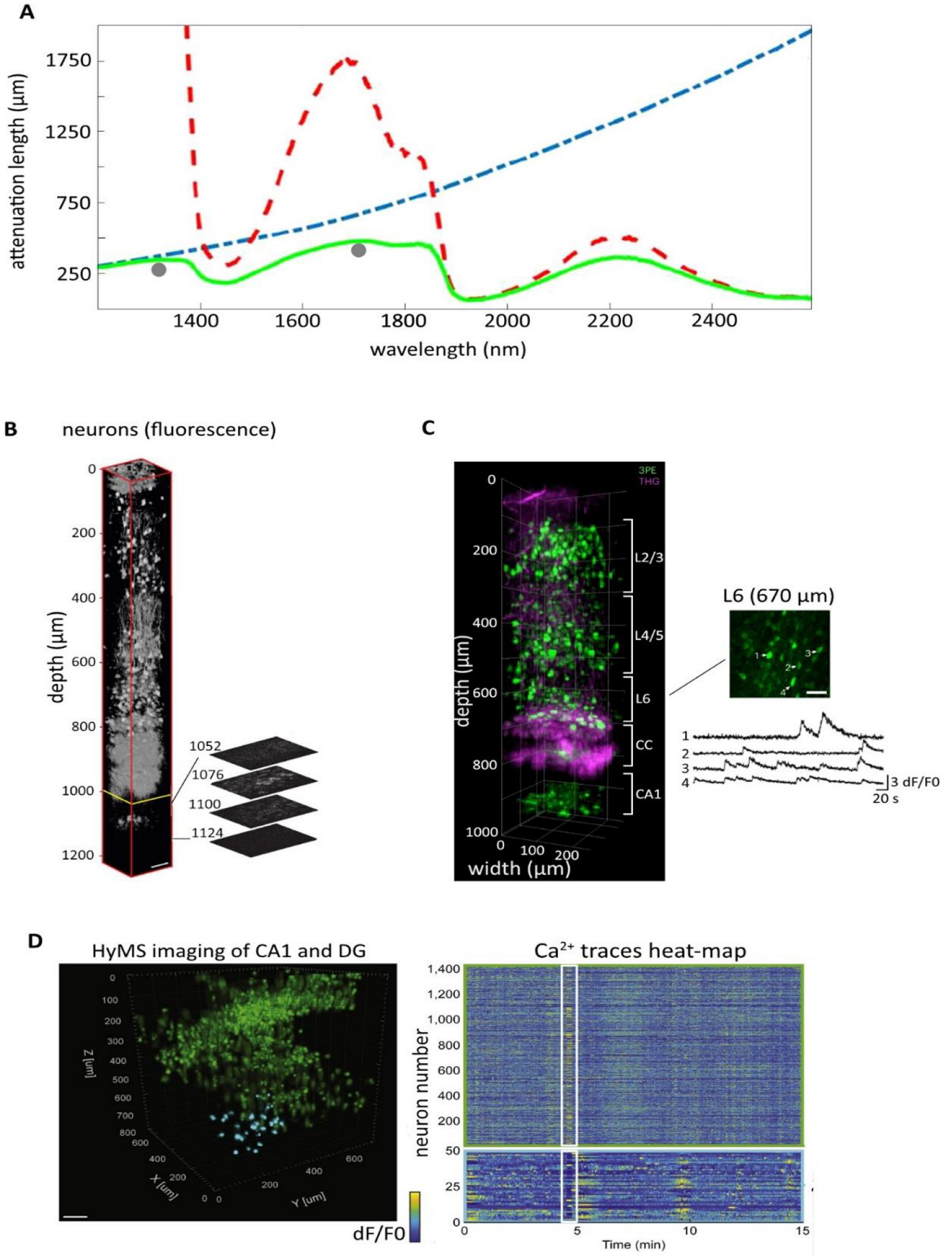
Deep within the brain: three-photon microscopy. (A) Attenuation spectrum of a tissue model based on Mie scattering theory and water absorption showing wavelength-dependent water absorption length (*l*_a_, red-dashed line), scattering length (*l*_s_, blue-dashed line), and the combined effective attenuation length (*l*_e_, green solid line) (*l*_e_ = (1/*l*_a_ + 1/*l*_s_)^−1^). (B) Reconstruction of three-dimensional images obtained from 3P imaging of pyramidal neurons in mouse neocortex. Deeper frames (>992 μm) were normalized to the frame at 1076 μm depth. Other frames were normalized individually. Right images are optical sections representing fluorescence images of the stratum pyramidal. Scale bar, 50 μm. (C) In vivo 3P miniature microscopy of GCaMP6s-expressing neurons in different cortical layers and hippocampal CA1. The image is obtained from reconstruction of a 970 μm stack. Green depicts the GCaMP6s fluorescence extracted from 3P excitation. Traces on the right show calcium transients of GCaMP6s expression neurons (*n* = 4). L2/3, cortical layer 2/3; L4/5, cortical layer 4/5; L6, cortical layer 6; and CC, corpus callosum. Scale bar, 50 μm. D) Left: reconstruction of three-dimensional image from GCaMP6f-expressing hippocampal neurons obtained from a 15 min HyMS recording. Right: heatmap shows calcium transients traces of 1456 active neurons with zoom-in of example of traces in white rectangles. Scale bar, 100 μm. Image credits: (A) Adapted with permission from ref (98). Copyright 2019 American Chemical Society. (B) Reproduced with permission from ref (97). Copyright 2013 Springer Nature Limited. (C) Adapted with permission from ref (103). Copyright 2023 The Authors, under exclusive license to Springer Nature America, Inc. (D) Reprinted with permission from ref (102). Copyright 2019 Elsevier Inc. (http://creativecommons.org/licenses/by/4.0/).

As discussed above, one of the fundamental limitations in deep brain 2P imaging is the scattering and absorption of ballistic photons by brain tissues. The loss of ballistic photons due to the scattering results in weaker fluorescence signals from the focal plane and reduces the signal-to-background ratio (SBR) for deeper brain tissues. Instead of increasing the optical power, which can damage brain tissues, one strategy to reduce the attenuation of excitation photons and increase the depth of recording is to employ longer excitation wavelengths.^95,96^ Accounting for both tissue scattering and absorption effects, it has been shown that the optimal spectral ranges for 3P excitation in terms of brain tissue penetration are near 1300 and 1700 nm (Figure 5A).^97,98^ Compared to 2P, 3P excitation provides significant improvement in overall spatial resolution by enlarging the SBR and reducing the out-of-focus background. 3P excitation was first demonstrated in the 1990s^96^ and its first in vivo application enabled imaging from hippocampal neurons at a depth of 1.3 mm (Figure 5B).^97^ Since then, several improvements have been made to enhance the performance of 3P imaging. For example, these improvements provide the possibility of recording from cortical brain networks (up to 500 μm) with cellular and subcellular resolution through intact skull and without implanting a cranial window.^99^ Applying adaptive excitation source, 3P microscopy enables high temporal resolution recording from subcortical regions with a reduced laser power requirement (at 30 Hz with 35 mW).^100^ Furthermore, development of multiplexed sculpted light microscopy (HyMS) that combines 2P and 3P excitation, permitting volumetric Ca^2+^ imaging of 12000 neurons in cortical and subcortical regions at 1 × 1 × 1.22 mm^3^ volumes and at up to 17 Hz (Figure 5D).^101,102^ Finally, recent head-mounted miniature 3P microscopy is demonstrated to capture neuronal activity of freely behaving animal across the entire cerebral cortex and dorsal hippocampus (CA1), up to 1.2 mm depth and at a safe laser power (Figure 5C).^103,104^ While 3P microscopy offers several advantages, especially for deep brain imaging, several considerations should be taken into account in its application. For example, 3P imaging from GECIs using 1700 nm excitation wavelength can be challenging; red-shifted GECIs tend to have lower sensitivity over the long-term expression, and it has been shown that in deeper layers of cortex, their signals are weaker than GCaMP versions.^105,106^ The maximum excitation power is constrained by laser-induced linear thermal and nonlinear effects. Increasing power or continuous heating can result in higher temperatures and thus affect neural signaling and behavior. Additionally, nonlinear effects are caused by high intensities (and not average power), leading to more damaging consequences such as tissue ablation, disruption, or even cell apoptosis. The high intensity of 3P excitation pulses can also saturate fluorophores and affect imaging quality. Further improvements such as red-shifted fluorescence indicators or commercial 3P laser optimization are required for deeper brain imaging with higher temporal resolution. The existing commercial 3P laser systems often face challenges related to power output and compatibility with specific fluorophores. Overcoming these limitations would significantly expand the capabilities of 3P microscopy in brain science study.

## Large-Scale Image Analysis

In vivo multiphoton imaging of neuronal networks generates large amounts of data collected during imaging sessions. Each frame of the imaging data contains information about the activity of individual neurons or neuronal ensembles which contributes to the overall size of the data set. These large data sets can pose several challenges for data management and analysis. In this section, we present a summary of the general processes involved in analyzing GECI-based images obtained from 2P recording. Additionally, we outline commonly used analytical platforms for this type of analysis. We do not discuss the image processing algorithms in detail or downstream analyses of the data. Regardless of the project aims, experimental condition, or analytical demands, series of preprocessing steps are required to extract signals from changes in fluorescence intensity of the neurons expressing genetically encoded fluorescent indicators. These preprocessing steps generally include motion correction, segmentation into regions of interest (ROIs), and extraction of signals from ROIs. Motion artifacts may occur during recording sessions due to various sources such as animal movement, drift in imaging setup, or mechanical vibrations. These artifacts can cause changes in fluorescence intensity, which not only distort signals related to neuronal activity, but can also be misinterpreted as neuronal or brain activity itself.^68,107^ Several motion correction methods have been developed ranging from rigid transformations to fast nonrigid registration algorithms.^108,109^ These algorithms mainly realign the captured frames to a fixed reference image. For example, in rigid transformations algorithms, template matching via cross-correlation or phase correlation is commonly used to estimate shifts between frames and a reference image.^108,110^ Nonrigid registrations algorithms are typically derived by subdividing the FOV into multiple overlapping blocks and then interpolating the shifts obtained for each block at subpixel resolution. This approach enables correction for more complex distortions such as deformations within the FOV.^109,110^ ROI segmentation is a process that delineates the areas of interest, representing individual neurons (here) or neuronal populations from non-neuronal elements or surrounding noises. ROIs should encompass a set of pixels that are assumed to provide spatiotemporally coherent fluorescence signals, reflecting the activity of the underlying neurons. Various segmentation techniques, including manual selection, thresholding, and supervised and unsupervised computer vision algorithms can be employed to discriminate ROIs based on their fluorescence characteristics and spatial distribution in the captured image.^75,111,112^ After segmentation, the resulting data undergo a signal extraction process to evaluate the temporal dynamics of fluorescence intensity within each ROI over time, reflecting changes in intracellular Ca^2+^ concentration associated with neuronal activity. The simplest method for this extraction is to average the fluorescence intensity across all pixels belonging to each respective ROI. While this method can be effective, it has some limitations; the extracted signal can be contaminated by several sources including neuropil and fluorescence from adjacent neurons. This can lead to inaccuracies in quantifying neuronal activity within the ROI.^26,68,112^ Various advanced methods, such as joint modeling or simultaneous spatial-temporal decomposition, have emerged to overcome these challenges. For example, in joint modeling, the neuropil signal and neuronal activity signal are modeled simultaneously within each ROI. By incorporating both components into the model, this approach allows for separation and quantification of the neuronal signal from background fluorescence.^112,113^ In simultaneous spatial-temporal decomposition, several algorithms have been developed to simultaneously estimate the optimal footprint of sources and their corresponding temporal activity.^114^ This is especially important in densely GECI-labeled neurons, where individual sources may overlap. The achieved calcium traces should be then normalized to account for variations in baseline fluorescence intensity among ROIs, enabling comparisons of activity both within and across recording sessions. The most common approach is to measure the change in fluorescence intensity relative to the baseline fluorescence often denoted as Δ*F*/*F*.^114−116^ While this approach is widely used for GECI-based recording of neuronal activity, it should be noted that several factors can influence computed Δ*F*/*F* values during calcium transients. These include recording parameters, the type of GECI, the brain region and cell type of interest, the expression level of GECI, and the magnitude of activity in the targeted neuron. To ensure data integrity, it is important to exclude Δ*F* and *F* values below the background noise level. One common method here is setting a threshold based on statistical measures.^26,114,116^ For example, neurons lacking calcium transients exceeding three standard deviations above their mean background fluorescence intensity may be excluded from further analysis.

Several programming languages, including Python and MATLAB, offer various image processing functions and libraries. In addition, numerous open-source resources are available on platforms such as GitHub that often include scripts and pipelines developed to address specific experimental needs or for specific data set analysis (based on their developer requirements). The compatibility of these customized codes is not always guaranteed; they generally demand adaptation/modification to serve specific experimental/imaging setups or data characteristics and thus influences their practicability for different laboratories or researchers. More user-friendly algorithms are now available for GECI-based image analysis. CalmAn,^117^ EZcalcium,^118^ and Suite2p^119^ are among the most popular data processing pipelines, offering user-friendly interface and efficient algorithms for image analysis. As the number of research laboratories utilizing multiphoton microscopy continues to rise, the demand for constant maintenance and updating of software packages by the developers also increases. Lastly, it is worth noting that researchers also have access to massive and open databases of large-scale imaging data provided by institutions like Allen Brain Map in Allen Institute (https://portal.brain-map.org/) or the International Brain Lab (https://www.internationalbrainlab.com). For example, the Allen Brain Observatory provides massive amounts of information on in vivo physiological activity in the mouse brain based on in vivo 2P calcium imaging from several brain regions during visual stimuli. Advanced analysis modules dedicated for these large-scale data are also provided by these institutions, shaping a promising future for in vivo multiphoton imaging of brain networks.

## Conclusion

In vivo multiphoton microscopy has opened a new window into the brain and revolutionized our understanding of the brain by providing new insights into the neuronal correlates of behavior. With all the advantages and challenges mentioned above, the current state-of-the-art technology provides access to living brain networks at the level of individual neurons, neuronal ensembles, and circuits that were unattainable in the past. Further innovations in multiple disciplines, such as optics, genetics and protein engineering, computer science and machine learning, and computational neuroscience, will likely improve temporal resolution and provide access to deeper brain structures and larger field of views. Furthermore, the increasing number of neuroscience research laboratories interested in employing this technology will drive the quest for integrating sophisticated systems into more user-friendly optical instrumentation. Lastly, the ongoing advances could provide the possibility to simultaneously record brain networks at multiscale, from genes to brain regions, with the ultimate goal of decoding brain function.
